# Assessment of interfraction dose variation in pancreas SBRT using daily simulation MR images

**DOI:** 10.1007/s13246-023-01324-6

**Published:** 2023-09-25

**Authors:** Tony Young, Mark Lee, Meredith Johnston, Theresa Nguyen, Rebecca Ko, Sankar Arumugam

**Affiliations:** 1 Liverpool and Macarthur Cancer Therapy Centres, Sydney, Australia; 2 Ingham Institute, Sydney, Australia; 3 Institute of Medical Physics, School of Physics, University of Sydney, Sydney, Australia; 4 South Western Sydney Clinical School, University of New South Wales, Sydney, Australia

**Keywords:** Interfraction, Dose variation, Pancreas

## Abstract

Pancreatic Cancer is associated with poor treatment outcomes compared to other cancers. High local control rates have been achieved by using hypofractionated stereotactic body radiotherapy (SBRT) to treat pancreatic cancer. Challenges in delivering SBRT include close proximity of several organs at risk (OARs) and target volume inter and intra fraction positional variations. Magnetic resonance image (MRI) guided radiotherapy has shown potential for online adaptive radiotherapy for pancreatic cancer, with superior soft tissue contrast compared to CT. The aim of this study was to investigate the variability of target and OAR volumes for different treatment approaches for pancreatic cancer, and to assess the suitability of utilizing a treatment-day MRI for treatment planning purposes. Ten healthy volunteers were scanned on a Siemens Skyra 3 T MRI scanner over two sessions (approximately 3 h apart), per day over 5 days to simulate an SBRT daily simulation scan for treatment planning. A pretreatment scan was also done to simulate patient setup and treatment. A 4D MRI scan was taken at each session for internal target volume (ITV) generation and assessment. For each volunteer a treatment plan was generated in the Raystation treatment planning system (TPS) following departmental protocols on the day one, first session dataset (D1S1), with bulk density overrides applied to enable dose calculation. This treatment plan was propagated through other imaging sessions, and the dose calculated. An additional treatment plan was generated on each first session of each day (S1) to simulate a daily replan process, with this plan propagated to the second session of the day. These accumulated mock treatment doses were assessed against the original treatment plan through DVH comparison of the PTV and OAR volumes. The generated ITV showed large variations when compared to both the first session ITV and daily ITV, with an average magnitude of 22.44% ± 13.28% and 25.83% ± 37.48% respectively. The PTV D95 was reduced by approximately 23.3% for both plan comparisons considered. Surrounding OARs had large variations in dose, with the small bowel V30 increasing by 128.87% when compared to the D1S1 plan, and 43.11% when compared to each daily S1 plan. Daily online adaptive radiotherapy is required for accurate dose delivery for pancreas cancer in the absence of additional motion management and tumour tracking techniques.

## Introduction

Pancreatic Cancer is associated with poor treatment outcomes compared to other cancers. Most pancreatic cancer patients present with locally advanced or metastatic disease, with tumours often unresectable due to local invasion of adjacent structures. For patients with resectable disease, the 5 year overall survival is less than 25% [1].

The use of hypofractionated SBRT (1–5 fractions) to treat pancreatic cancer has resulted in high local control rates [2–4]. Radiotherapy for pancreatic cancer is challenging due to the close proximity of several organs at risk (OARs) to the pancreas, such as the duodenum, small bowel and stomach. In addition to this, the target volume may have large inter and intra fraction variations due to body contour changes, internal organ motion and changes, and respiratory motion [5]. Respiratory motion alone has the potential to produce intra fraction target motion of the order of 10–20 mm [6]. Day to day interfraction motion can produce variations and displacements of greater than 10 mm for surrounding duodenum, small bowel and stomach in pancreatic cancer radiotherapy [7, 8]. Magnetic resonance guided radiotherapy has also improved the potential of online adaptive radiotherapy for pancreatic cancer [9], with MRI providing high resolution images with superior soft tissue contrast compared to CT [10]. This soft tissue contrast is necessary as there may be considerable interfraction position variation of the target and organs at risk relative to bony anatomy [11, 12], and aids in target delineation, resulting in smaller target volumes and reducing interobserver variation [13].

The advent of adaptive treatment planning has also been required to counter the intra fraction motion within the abdomen which may affect the delivery of radiation to pancreatic cancer. There have been studies attempting to quantify allowable motion in pancreatic cancer radiotherapy treatment, and the clinical effects due to the inter and intra fraction motion [5, 11, 14–16].

With the introduction of hybrid MR-guided Linac (MRg-linac) systems, the ability to deliver online MR guided adaptive radiotherapy has become more common [9, 17–21]. These systems facilitate the adaption of treatment plans to accommodate daily position variations, however have strict time constraints as the patient remains on the table in the treatment position whilst plan adaptation occurs. These systems still require treatment planning to occur on a reference CT scan however, with the daily adaptation of treatment plan based off the change in treatment volumes and OAR volumes from the captured MRI guidance scan [22, 23].

The aim of this study was to investigate the variability of target and OAR volumes for different adaptive treatment regimens for pancreatic cancer, and to assess the suitability of utilizing a treatment day MRI for treatment planning purposes, relevant for centres with access to MRI but not an MRg-linac.

## Methodology

Ten volunteers were scanned on a Siemens (Erlangen, Germany) Skyra 3 T MRI with a flat radiotherapy couch and coil mounts. Volunteer ages ranged from 25 to 47 with a median age of 35 and body mass index (BMI) ranged from 19.7 to 29.0 with a median BMI of 24.0. Volunteers were scanned in full body vacuum bags for all sessions for setup consistency. Each volunteer was scanned in two sessions per day over 5 days to simulate a potential pancreas SBRT treatment regime, with a daily simulation scan for treatment planning, and a pre treatment scan for treatment. The second imaging session for each day was approximately three hours after the first imaging session. Volunteers were given instructions allowing only one cup of liquid and a small snack between scans, though compliance with these instructions was not strictly enforced being a volunteer study.

Each MRI scanning session consisted of the following scans as presented in Table 1—T1 weighted transverse VIBE with DIXON (16 s exhale breath hold), T2 weighted interleaved TruFISP (3 orthogonal planes) (1 min CINE) and a T1 weighted transverse 4D-MRI (5–6 min). Only the VIBE scan and the 4D MRI scan were used for this study.

**Table 1:** MRI scanning session sequences, time and use

Scan	Use	Time
T1 weighted transverse VIBE with DIXON (16 s exhale breath hold)	Anatomical contouring, treatment planning	~ 20 s
T2 weighted interleaved TruFISP (3 orthogonal planes) (1 min CINE)	2D motion assessment	~ 1 min
T1 weighted transverse 4D-MRI	3D motion assessment, ITV generation	~ 5–6 min

All scans were contoured and planned in the Raystation (RaySearch Laboratories, Stockholm, Sweden) Treatment Planning system (version 10b). The water only images from the T1 weighted VIBE Dixon MRI sequence were used for treatment planning. The first imaging session of the first day (D1S1) was considered the primary planning images for each volunteer. A Radiation Oncologist (RO) contoured a hypothetical tumour volume in the head region of the pancreas, with the last 2 cm of the pancreas used as the gross tumour volume (GTV) volume, as well as the nearby critical organs (ie stomach, duodenum). All other OARs required were contoured by radiation therapists (RTs) and physicists. The 4D MRI was used to generate an internal target volume (ITV) from the GTV, which covered the movement of the GTV over the volunteer breathing cycle. The ITV was transferred to the treatment planning dataset through registration of the exhale phase of the 4D-MRI scan. A 5 mm expansion was applied to the ITV to generate the PTV. The ITV generation using the 4D MRI sequence has been validated against CT previously [24].

Initially, each session on each day was registered to the primary planning images using a rigid registration which focused on the pancreas, vertebral body, and kidney ROIs. The ITV and nearby OARs were assessed for volume change, Dice similarity coefficient (DSC) and Hausdorff distance (HD) for each days mock treatment session compared to the primary planning images.

**Fig. 1:**
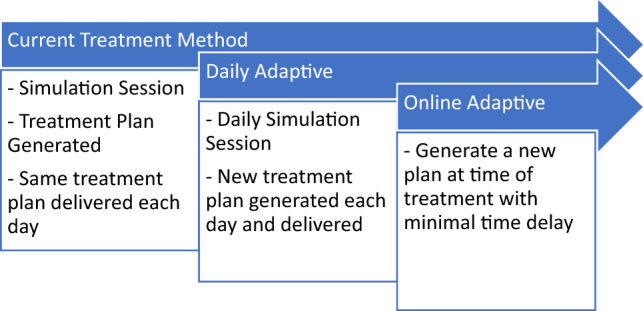
Summary of the different mock adaptive treatment methods considered in this study

Three different adaptive treatment methods were considered in this study and are presented in Fig. 1. To enable treatment planning on the MRI images, density overrides were applied to the external contour (1 g/cm^3^) and vertebral body (1.12 g/cm^3^) as per the International Commission on Radiation Units and Measurements (ICRU) Report 46 recommendations. The 6MV dual arc VMAT treatment plan was generated following the current departmental protocols on the primary planning images. These clinical goals have been taken from the MASTERPLAN clinical trial (ACTRN12619000409178) and are presented in Tables 2 and 3. This treatment plan was propagated via the rigid registration to all other imaging sessions and recalculated.

**Table 2:** Planning target volume clinical goals from the MASTERPLAN clinical trial

Parameter	Per protocol	Minor variation	Major variation
PTV40_EVAL D90%	> 100%	90–99%	< 90%
PTV40 D99%	> 30 Gy	25–30 Gy	< 25 Gy
ITV D99%	> 33 Gy	30–33 Gy	< 30 Gy
Max Dose (D0.5 cc)	110–130%	130–140%, OR < 110%	> 140%

**Table 3:** OAR clinical goals considered for this study, taken from the MASTERPLAN clinical trial

Organ	Constraint	Per protocol	Minor variation	Major variation
Duodenum	Dmax (0.5 cc)	< 33 Gy	≤ 35 Gy	> 35 Gy
	V30	< 5 cc	5–10 cc	> 10 cc
Stomach	Dmax (0.5 cc)	< 33 Gy	≤ 35 Gy	> 35 Gy
	V30	< 5 cc	5-10 cc	> 10 cc
Small bowel	Dmax (0.5 cc)	< 33 Gy	≤ 35 Gy	> 35 Gy
	V30	< 5 cc	5-10 cc	> 10 cc
Combined kidneys	V12Gy	< 25%	25–30%	> 30%
Liver	V12Gy	< 40%	≤ 50%	> 50%

The first imaging session of each day (S1) was also replanned as would occur with a daily adaptive treatment plan, and corresponding treatment (S2) later that day. Treatment plan quality consistency was maintained through the use of templates for VMAT optimisation objectives and comparison of clinical goals in the initial D1S1 treatment plan. This method was utilised as it was the potential clinical translation of this work, with patients undergoing a daily MRI simulation session followed by radiotherapy on a conventional linear accelerator. The treatment plan generated on the first session of each day was propagated to the second session and recalculated, with this accumulated to indicate daily replanning and treatment. In addition, a treatment plan was generated on the second session of each day, with these accumulated to indicate online treatment adaptation. This was completed to ensure that the clinical goals were able to be met on that dataset. Both instances were compared with the treatment plan generated on the primary planning images through DVH comparison of the clinical goals presented in Tables 2 and 3.

## Results

**Fig. 2:**
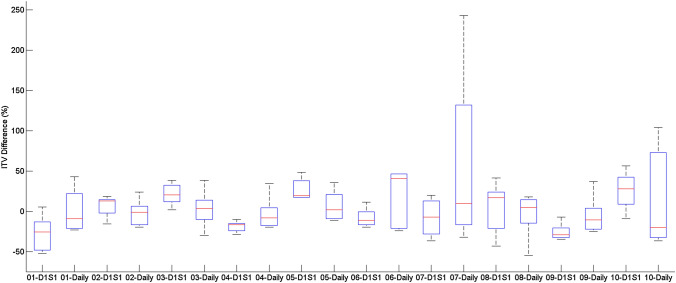
ITV variations for each volunteer considering a reference (D1S1) scan or a daily reference (Daily) scan

The ITV varied across all volunteers and for both sets of comparisons, with the ITV variations for each volunteer presented in Fig. 2. On average, the ITV variation was − 0.85% ± 23.68% across all volunteers. ITV variations over the course of the mock treatment were quite high for both comparison with the baseline scan and the daily scan for each volunteer.

Contour assessment was also undertaken considering the GTV, ITV and the surrounding organs at risk for the second imaging session of each day for both when a single reference scan is considered or a daily reference scan. The results for the average across the 10 volunteer dataset, considering the DSC, mean HD agreement and the volume change for both comparisons is shown in Table 4. The DSC results were improved when considering a daily reference as opposed to a single reference for all volumes considered. When the volume change is considered, the absolute volume changes are also smaller when a daily reference is considered. The external volume DSC is similar for both when compared to a D1S1 scan and to a daily scan, with a result of 0.959 ± 0.021 and 0.968 ± 0.009 respectively. All OARs had poor DSC, as well as large amounts of volume change for all comparisons.

**Table 4:** Contour comparison results when considering a single reference scan (D1S1) or a daily reference scan (daily)

	External	GTV	ITV	Duodenum	Pancreas	Small bowel	Stomach
VS D1S1 volumes							
Average dice	0.959	0.521	0.594	0.542	0.686	0.569	0.654
StDev dice	0.021	0.152	0.158	0.097	0.087	0.206	0.073
Average Hausdorff	11.39	3.74	3.66	4.55	2.89	10.18	6.63
StDev Hausdorff	3.442	1.550	1.685	1.515	0.780	9.286	2.492
Average volume CHANGE	0.20%	− 5.90%	− 0.25%	− 4.44%	3.30%	73.86%	− 9.68%
StDev volume change	2.61%	16.20%	19.70%	24.19%	20.01%	217.14%	43.76%
Average absolute volume change	1.78%	14.33%	16.56%	19.66%	12.79%	91.02%	39.14%
StDev absolute volume change	1.82%	8.54%	9.14%	13.32%	15.19%	209.77%	17.79%
VS daily S1 volumes							
Average dice	0.968	0.538	0.615	0.585	0.722	0.653	0.704
StDev dice	0.009	0.139	0.141	0.108	0.061	0.071	0.076
Average Hausdorff	10.935	3.476	3.411	4.034	2.576	7.096	5.137
StDev Hausdorff	3.332	1.595	1.666	1.829	0.647	1.693	1.313
Average volume change	0.41%	4.50%	8.00%	− 3.78%	− 2.89%	3.55%	1.29%
StDev volume change	0.94%	21.97%	18.95%	10.49%	7.93%	15.08%	41.80%
Average absolute volume change	0.88%	14.49%	11.45%	8.62%	6.63%	13.32%	31.42%
StDev absolute volume change	0.46%	16.49%	16.86%	6.58%	4.83%	6.66%	25.53%

The average DSC, HD and volume changes for various volumes are presented

**Fig. 3:**
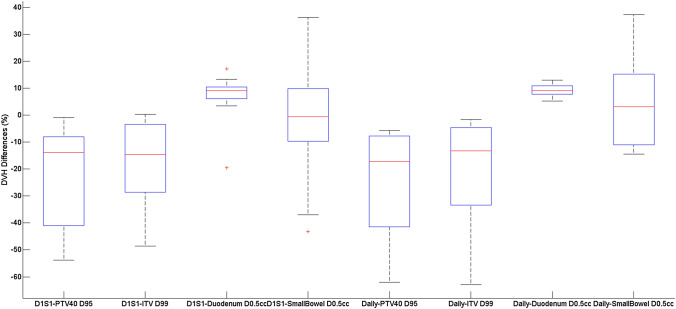
DVH differences for PTV D95, ITV D99, Duodenum D0.5 cc and small bowel D0.5 cc considering the D1S1 reference plan comparison and the Daily reference plan comparison across all volunteers

Table 5 displays the mock treatment plan comparison as a whole, with each dose accumulation methodology averaged. Figure 3 displays the average results for the rigid dose accumulation for both comparison to the D1S1 plan and the daily plan. Online adaptive generally allowed clinical goals to be met, though large variations are seen for the small bowel and stomach V30 for the online adaptive comparison due to the small absolute volumes receiving 30 Gy for these OAR in the treatment plans.

**Table 5:** Dose accumulation summary considering the different scenarios. These DVH parameters have been averaged for all volunteers

DVH statistics	Rigid accumulation compared to D1S1 plan (%)	Rigid accumulation compared to daily plan (%)	Online adaptive (%)
PTV40 D95	− 23.31	− 23.29	1.67
PTV40 D99	− 33.86	− 33.58	0.87
PTV40_EVAL D90	− 20.56	− 19.93	− 0.73
ITV D99	− 17.68	− 19.69	1.49
DUODENUM V30	1.48	53.91	− 2.21
DUODENUM D0.5 cc	6.21	9.29	− 2.16
SMALLBOWEL V30	128.87	43.11	149.22
SMALLBOWEL D0.5 cc	− 1.47	7.66	− 8.91
STOMACH V30	967.47	9.42	156.77
STOMACH D0.5 cc	− 4.64	60.26	− 24.86
LIVER D50	− 4.31	14.48	− 13.26
COMB_KIDNEYS V12	37.04	22.84	64.62

The target volume coverage on average was reduced when considering the accumulated dose for both mock treatment with either a D1S1 reference plan or a daily reference plan as can be seen in Fig. 3. The PTV40 D95 was on average reduced by − 23.3% when both sets of reference plans are considered, with small bowel and duodenum V30 increasing for both sets of plan comparison. The duodenum V30 increased by 1.48% compared to the D1S1 reference plan, and 53.91% compared to the daily reference plan, while the small bowel V30 increased by 128.87% and 43.11% respectively.

**Fig. 4:**
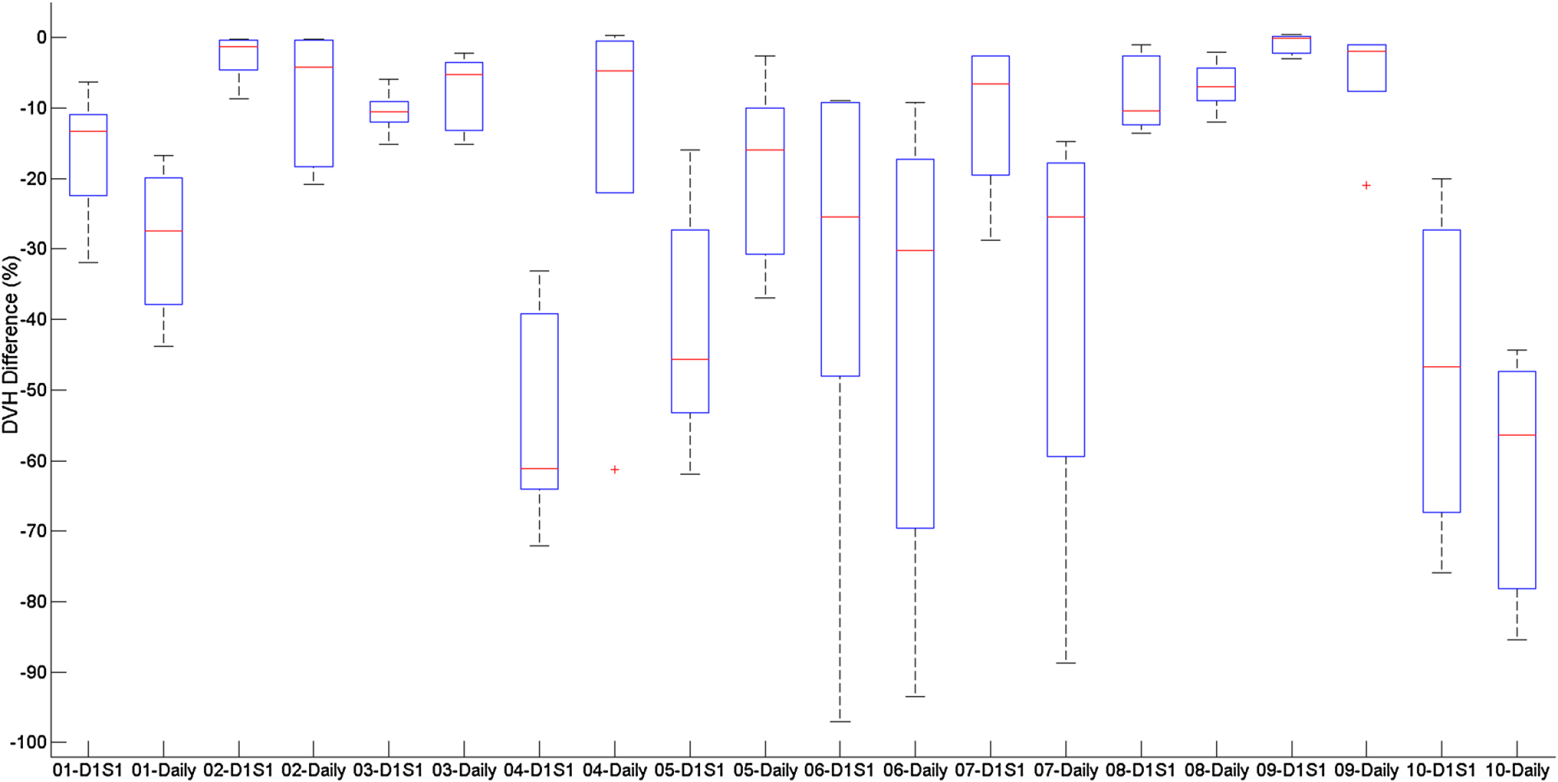
DVH differences for PTV D95 for all plan comparisons

**Fig. 5:**
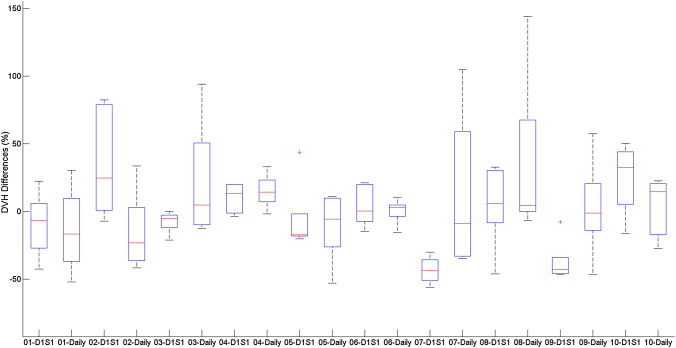
DVH differences for small bowel D0.5 cc for all plan comparisons

Figures 4 and 5 display the DVH differences for the PTV40 D95 and the small bowel D0.5 cc for both sets of plan comparisons for all volunteers. On a per volunteer basis, these differences varied between plan comparisons, with some volunteers benefitting from the daily plan adaption. Though on average, as seen in Fig. 3, these parameters may show similar variations, the differences are much more varied on a per volunteer basis.

## Discussion

This study considered the variation in internal anatomy between daily imaging sessions and over different days, and the variation in dose to target volume and nearby organs at risk for a simulated pancreas SBRT radiotherapy treatment. MR imaging was used for this comparison, with volunteers undertaking twice daily MRI simulation scans over 5 days within a 2 week period on a radiotherapy MRI simulator to simulate a 5 fraction treatment regime, with the first imaging session of each day a simulation session, and the second imaging session a mock treatment session, ie pre treatment imaging. A limitation of this study is that it is a volunteer study considering only a simulated tumour in the head of the pancreas and does not contain any patient data where the tumour location may vary.

The variation in internal volume anatomy has been studied previously. Heerkens et al. [25] studied MRI based tumor motion characterization, using sagittal and coronal cine MRIs of 60 s in 15 pancreatic cancer patients to quantify tumor motion. This study found tumor motion was largest in the craniocaudal direction, with an average amplitude of 15 mm and with a range of 6–34 mm. The average in the anterior–posterior direction was 5 mm, with range 1–13 mm, and in the lateral direction an average of 3 mm, range 2–5 mm. Alam et al. [26] looked at a pre treatment, verification and post treatment MRI for each abdominally compressed pancreas cancer patient treatment fraction, finding median (max) interfraction deformation for the stomach/duodenum and small bowel of 6.1 (25.8) mm and 7.9 (40.5) mm respectively and median intrafraction deformation was 5.5 (22.6) mm and 8.2 (37.8) mm respectively. These large variations are similar for both inter and intra fraction motion, indicating large displacements for both even with abdominal compression. This study also reported median DSC similarity scores for the duodenum-stomach and small bowel of 0.7, which is similar to that reported in this study.

It had been considered that a daily replanning exercise may be sufficient for this cohort of patient, especially in the absence of an MR guided linac. From Table 2, the average DSC, HD and volume change had improved results for all volumes compared in the daily scans when compared to the results which compared the volumes back to the D1S1 reference scan. However the dosimetric results from Table 5 for the daily plans recalculated on the treatment scan of the day showed large variations in dose coverage and OAR dose. Poor compliance with instructions for food and drink between imaging sessions may have contributed to the large variations in organ location and volume within this study for the daily replan. Additionally, this cohort of patients would potentially be treated with an empty stomach each day for consistency, which did not occur in this volunteer study. This may have contributed to the day to day variations seen between scans. Some volunteers would have benefited from the daily scans though, as can be seen from the comparisons in Figs. 4 and 5 regarding the PTV D95 and the OAR DVH differences, with approximately 40% of volunteers showing better agreement in regards to DVH analysis with the appropriate reference plan.

Large variations in the ITV were seen in Fig. 2 and Table 4. Additionally in Table 4, variations in volume were seen for the GTV. As the GTV was re-contoured for each imaging session, intra-observer contour variation may have contributed to the ITV variation as well, with this being a potential source of error [27, 28]. Additionally, a lack of motion management may also contribute to this ITV variation. This shows that a 4D ITV method may not be sufficient for these patients, with other motion management techniques [29, 30] such as gated techniques, implanted markers/tumour tracking or compression techniques able to reduce the variation in ITV on a daily basis, and potentially enable more accurate delivery of dose to the tumour whilst maintaining OAR doses [31, 32]. However this would not necessarily reduce any interfraction motion of the OAR, which occurred throughout this study for all volunteers. Fiducial markers implanted in the target volume would be useful for determining the interfraction and intrafraction motion of the target. Though this study was able to visualize the target volume, fiducial markers would aid the accuracy of the registration back to reference images and plan.

When comparing the dose delivery considering only a single baseline plan, as well as comparing to a daily plan, the target dose was deficient in most days considered due to anatomical changes. These variations included both nearby OAR variations, as well as changes in ITV generation due to the variability in volunteer breathing, which generated a different daily PTV volume for the day. It should be considered that if the ITV was smaller, that the previous dose coverage should still be sufficient for target coverage, however the average DSC of 0.594 ± 0.158, average HD of 3.66 mm ± 1.69 mm and average volume variation of − 0.25% ± 19.70% would indicate that the ITV volume and position varied when compared to the D1S1 ITV. For the daily plan, these results were only slightly improved, with an average DSC of 0.615 ± 0.141, average HD of 3.41 mm ± 1.67 mm and average volume variation of 8% ± 18.95%.

The conventional method of simulation and planning based off a single reference scan is not appropriate for this treatment site for a hypofractionated SBRT treatment as per the variations seen in this study. Scanning on the day and completing treatment planning with the scan on the day may be appropriate for some patients with appropriate motion management but still may have large variations in internal organ anatomy between the scan on the day and the treatment scan, particularly if strict protocols are not followed regarding intake. Online adaptive treatment is the best option for ensuring target dose coverage whilst minimizing dose to nearby OARs, particularly the duodenum, small bowel and stomach [33]. This was seen from the results presented in Table 5, though some large variations in OAR dose are presented due to the low absolute dose for these DVH parameters. This methodology is current best practice and has been utilized with hybrid MR-guided linacs [8, 19–21, 34], allowing delivery of hypofractionated SBRT treatment for pancreatic cancer using real time adaptation of the dose distribution to account for day to day variations in organ shapes and position. This is currently not possible with conventional linear accelerators however, and a daily MRI scan in conjunction with daily plan adaptation and additional motion management techniques and tracking may allow more accurate treatment of pancreas SBRT whilst reducing dose to nearby OARs.

## Conclusion

Daily online adaptive radiotherapy is required for accurate dose delivery for pancreas cancer in the absence of additional motion management and tumour tracking techniques.

## Data Availability

Due to the nature of this research, participants of this study did not agree for their data to be shared publicly, so supporting data is not available.

## Declarations

### Research involving humans and animal participants

Volunteer data was acquired with local ethics committee approval, HREC/15/LPOOL/506.
